# Data on numerosity discrimination, inhibition and arithmetic during the early school years

**DOI:** 10.1016/j.dib.2019.104062

**Published:** 2019-05-25

**Authors:** Stephanie A. Malone, Verena E. Pritchard, Michelle Heron-Delaney, Kelly Burgoyne, Arne Lervåg, Charles Hulme

**Affiliations:** a Institute for Learning Sciences and Teacher Education, Australian Catholic University, Australia; b University of Manchester, Australian Catholic University, UK; c Department of Education, University of Oslo, Norway; d University of Oxford, Australian Catholic University, UK

**Keywords:** Approximate number system (ANS), Arithmetic development, Numerosity judgment, Inhibition, Childhood, Magnitude comparison

## Abstract

Participants consisted of 496 children (mean age = 6 years; 9 months) recruited from 11 schools in Brisbane, Australia. Children were assessed on the addition and subtraction subtests of the *Test of Basic Arithmetic and Number Skills (TOBANS)*, an adapted version of the *Head-Toes-Knees-Shoulders task* to measure inhibition, numerosity discrimination using eight subtests varying ratio (2:3 or 5:6) and congruency, and non-verbal cognitive ability using an adapted version of *Raven's Coloured Progressive Matrices*. Information on children's demographics (gender, English as an additional language, and learning difficulty status) is also provided. All assessments were administered during the second year of formal schooling (i.e. Grade 1). Findings regarding the impact of inhibition on the relation between numerosity discrimination and arithmetic are reported elsewhere [1].

**Table:** Specifications Table

Subject area	*Psychology, Education*
More specific subject area	*Cognitive Development*
Type of data	*Text file (.csv)*
How data was acquired	*Individual and group assessment of children by research assistants and research fellows. All data was collected using paper and pencil methods. Numerosity discrimination stimuli were designed on an Apple Macbook Pro (15-inch), using Microsoft PowerPoint.*
Data format	*Raw data files*
Experimental factors	*Children who scored below chance (i.e. < 50% correct) on any numerosity discrimination condition were excluded since such scores are likely to reflect guessing only. Where scores are at or above chance they reflect the efficiency with which a child can make the discrimination required.*
Experimental features	*Individual assessment of children's inhibition and arithmetic performance, and group assessment of children's numerosity discrimination ability and non-verbal cognitive ability.*
Data source location	*Brisbane, Queensland, Australia.*
Data accessibility	*Data are within this article.*
Related research article	S. A. Malone, V. E. Pritchard, M. Heron-Delaney, K. Burgoyne, A. Lervåg, C. Hulme. The relationship between numerosity discrimination and arithmetic skills reflects the Approximate Number Sense and cannot be explained by inhibitory control. Journal of Experimental Child Psychology (2019), https://doi.org/10.1016/j.jecp.2019.02.009[1]

**Table:** 

**Value of the data** • May be used to investigate whether there are differences in the cognitive profile of children who experience difficulties in mathematics compared to typically developing children. • Amenable to the examination of whether the individual observed variables of numerosity discrimination differentially relate to arithmetic performance. • Available for comparison with effects gained with other alternative measures of inhibitory control. • Can be used to determine the stability of the relation between numerosity discrimination and arithmetic skills across development.

## 1 Data

The NumerosityArithmetic.csv data file provides the children's scores on each measure administered, as well as their age, gender, English as an additional language status, and learning difficulty status.

## 2 Experimental design, materials and methods

### 2.1 Participants

Participants include 496 children from 11 primary schools in Brisbane, Australia (241 boys, mean age = 81.23 months, *SD* = 4.25, range = 71–99 months). These children were all in their 2nd year of formal education (i.e. Grade 1). The age range is greater than expected due to a small minority of children previously repeating their Preparatory year. Additional information on the participants is available in Malone et al. [1].

### 2.2 Procedure

Ethical approval for this research was obtained from the Australian Catholic University Research ethics committee. Prior to assessment, the schools and parents/carers of the children provided consent. Children also provided their assent to participate.

Children were assessed across three testing sessions where they worked individually (one-to-one) with a researcher in a quiet area of the school, and once in group format where all participating children in a single class completed the assessments simultaneously. These assessments were administered during the latter half of the children's second school year (i.e. Grade 1). During these testing sessions children were also assessed on a range of additional non-relevant measures.

### 2.3 Measures

#### 2.3.1 Numerosity discrimination

This assessment consisted of eight sub-tests, each assessing children's ability to determine the more numerous dot array from a stimulus pair. The sub-tests systematically varied the ratio between stimulus pairs (2:3 or 5:6) and congruency between numerosity and dot surface area (super congruent, congruent, incongruent, super incongruent). Each subtest consisted of 40 trials (10 exemplars presented four times in a random order). Chance performance on these measures is 50% correct; scores for children who performed below chance on any condition were excluded since such scores are likely to reflect guessing only. Where scores are at chance or above they reflect the efficiency with which a child can make the discrimination required. The data file provides three variables per subtest: the number of correct trials (raw score), performance accuracy (i.e., correct trials/total trials attempted), and the performance score adjusted for accuracy (i.e. if less than 50%, scored as missing [-9999]).

#### 2.3.2 Inhibition

This two-part assessment used a modified version of the Head Toes Knees Shoulders task [2]. In Part 1, children were asked to touch their head or toes (6 trials); in Part 2 they were asked to touch their head/toes and shoulders/knees (14 trials). Two points were awarded if the child's initial response was towards the opposite body part (i.e., touched their head when asked to touch their toes); one point was awarded for self-corrections. The data file provides their total score across the two parts (max. 40).

#### 2.3.3 Arithmetic

Addition and subtraction abilities were assessed using the corresponding subtests from the Test of Basic Numeracy and Arithmetic Skills (TOBANS [3]). Scores for each subtest reflect the number of problems solved correctly in 1 minute.

#### 2.3.4 Non-verbal cognitive ability

A modified 12-item version of Raven's Coloured Progressive matrices [4] suitable for group administration was used. Scores on this task reflect the total number of items completed correctly.

Descriptive statistics (means, standard deviations) were calculated for each variable (Table 1). Note that the number of participants varies across the numerosity discrimination task; this is reflective of the different numbers of children that performed below chance in these conditions.

**Table 1:** Descriptive statistics for measures of numerosity discrimination, arithmetic and inhibition.

Task (max)	*N*	Mean (*SD*)	Range	Skewness	Reliability
Numerosity Discrimination					
2:3 Congruent (40)	430	19.72 (6.75)	2–40	.17	–
2:3 Incongruent (40)	412	16.82 (5.49)	3–36	.25	–
2:3 Super congruent (40)	436	21.86 (7.22)	1–40	.02	–
2:3 Super incongruent (40)	362	15.86 (5.96)	2–40	.55	–
5:6 Congruent (40)	419	14.82 (5.13)	3–31	.21	–
5:6 Incongruent (40)	365	12.67 (4.33)	2–26	.38	–
5:6 Super congruent (40)	420	16.67 (5.53)	3–40	.29	–
5:6 Super incongruent (40)	283	11.55 (3.94)	2–25	.44	–
Inhibition					
HTKS (40)	459	34.67 (5.51)	5–40	−2.27	.76
Arithmetic					
Addition (60)	474	12.32 (7.42)	0–58	1.90	.92^a^
Subtraction (60)	469	7.32 (4.33)	0–33	1.32	.88^a^
Non-verbal cognitive ability					
Ravens CPM (12)	467	8.15 (1.46)	2–11	-.64	.48

*Note.* Unless otherwise specified, all reliabilities are Cronbach's alpha. HTKS: Head–Toes–Knees–Shoulders; CPM: Coloured Progressive Matrices.

a Test-retest reported in the manual.
