# Supplementary material for: Data on numerosity discrimination, inhibition and arithmetic during the early school years
Source: Data Brief. 2019 May 25;25:104062. doi: 10.1016/j.dib.2019.104062 (PMC6558229; doi:10.1016/j.dib.2019.104062)
Supplement: Multimedia component 1 [file mmc1.docx]

**Author Declaration**

We wish to confirm that there are no known conflicts of interest associated with this publication and there has been no significant financial support for this work that could have influenced its outcome.

We confirm that we have given due consideration to the protection of intellectual property associated with this work and that there are no impediments to publication, including the timing of publication, with respect to intellectual property. In so doing we confirm that we have followed the regulations of our institutions concerning intellectual property.

We further confirm that any aspect of the work covered in this manuscript that has involved either experimental animals or human patients has been conducted with the ethical approval of all relevant bodies and that such approvals are acknowledged within the manuscript.

Best wishes,

Stephanie Malone.
